# Alteration in the time and/or mode of delivery differentially modulates early development in mice

**DOI:** 10.1186/s13041-020-00578-5

**Published:** 2020-03-09

**Authors:** Morgane Chiesa, Diana C. Ferrari, Yehezkel Ben-Ari

**Affiliations:** Fundamental Research Department, Neurochlore, Ben-Ari Institute of Neuroarcheology (IBEN), Marseille, France

**Keywords:** Cesarean section delivery, Preterm birth, Eye opening, Neurodevelopmental disorders, Ultrasonic vocalizations

## Abstract

Delivery is a complex biological process involving hormonal and mechanical stimuli that together condition the survival and development of the fetus out of the womb. Accordingly, changes in the time or way of being born are associated with an alteration of fundamental biological functions and hypothesized to promote the emergence of neurodevelopmental disorders. Hence, the steadily rise in preterm birth and cesarean section (CS) delivery rates over the past years has become a worldwide health concern. In our previous work, we reported that even though no long-term autistic-like deficits were observed, mice born preterm by CS presented early transient neuronal and communicative defects. However, understanding if these alterations were due to an early birth combined with CS delivery, or if prematurity solely could lead to a similar outcome remained to be evaluated. Using mice born either at term or preterm by vaginal or CS delivery, we assessed early life ultrasonic vocalizations and the onset of eye opening. We report that alterations in communicative behaviors are finely attuned and specifically affected either by preterm birth or by the association between CS delivery and preterm birth in mice, while delayed onset of eye opening is due to prematurity. Moreover, our work further underlies a gender-dependent vulnerability to changes in the time and/or way of being born with distinct outcomes observed in males and females. Thus, our results shed light on the intricacy of birth alterations and might further explain the disparities reported in epidemiological studies.

## Introduction

Delivery is a complex process that, in mammals, involves the cooperation of hormonal and mechanical stimuli originating from both the mother and the fetus. These mechanisms are essential for vital adjustments to swiftly take place to allow the survival of the fetus and its adaptation to extrauterine life. Indeed, crucial mechanisms are initiated at birth including the activation of the gluconeogenesis pathway to promote a high-fat and low-carbohydrate diet [1] and the triggering of thermogenesis [2]. The fetus’ brain is also prepared for this transition by slowing down its growth right before birth [3] and dampening its sensibility to pain [4]. Moreover, preclinical studies have recently defined birth as a critical period that initiates the development of brain structures such as the formation of sensory maps [5, 6]. For these reasons, changing the time and/or way of being born engender deviations from the original developmental program that may lead to short- and/or long-term consequences [7].

Cesarean section (CS) delivery and preterm birth, two common birth modifications, increase the risk of neonatal morbidity [8, 9], and babies born in these conditions present wide-ranging shortcomings including cardiovascular defects [10, 11], gluconeogenesis deficiency [12, 13], and inadequate lung activity [14, 15]. In addition, preterm birth and CS delivery have been associated with an increased risk of developing pathological conditions including autoimmune diseases [16, 17], obesity [18], and neurodevelopmental disorders such as autism spectrum disorders (ASD) [19, 20], thus explaining why their worldwide increasing rates [21, 22] are becoming major health concerns. However, these associations remain controversial since some studies have attributed no relationship between preterm birth/CS delivery and the incidence of neurodevelopmental disorders [23, 24]. These contradictory findings might emerge from the difficulty to embrace the heterogeneity of parameters associated with birth, highlighting the need of preclinical studies to understand the intricate impact of perinatal factors, sole or joint, on postnatal development.

Recently, we reported that even though mice born by CS delivery did not exhibit long-term ASD-like features, neonatal pups presented transient alterations in neuronal morphology and deficits in communicative behaviors that were aggravated by the time of birth [25]. The question still remained to determine if these early developmental defects were due to the association of CS delivery with prematurity, or to prematurity per se. In that aim, we assessed early communicative behaviors to complement our previously published results since alterations in neonatal isolation-induced ultrasonic vocalizations participate in the characterization of many neurodevelopmental disorders including ASD [26]. In addition, we evaluated a developmental milestone, the onset of eye opening, since preterm birth has been associated with a delayed maturation of sensory functions [27, 28]. Moreover, a delayed onset of eye opening is observed in diverse perinatal pathological conditions [29–32], suggesting it as a critical factor to evaluate early life insults.

Here we report that ultrasonic vocalizations parameters are differentially modulated by preterm birth and CS delivery, while preterm birth independent of the mode of delivery induces a delayed onset of eye opening. Interestingly, these modifications further uncovered a gender-dependent vulnerability, with birth alterations affecting more strongly males than females. Altogether, these results suggest that the influence of birth-related modifications on general and brain developmental patterns is complex and might underlie the disparities reported in epidemiological studies.

## Materials and methods

### Animals

Swiss mice (Janvier Labs, France) were maintained on a 12:12-h light/dark cycle with ad libitum access to food and water. Mice were mated overnight by placing 2 females with one male and separated at 7 am on the next morning after checking for vaginal plugs. The day of vaginal plug detection was designated as embryonic day 0.5 (E0.5) and the gestation lasted for 19 days. Neonatal pups of both sexes were evaluated in four experimental groups: term vaginal (control group, *n* = 36 (13 males and 23 females) from 4 litters), preterm vaginal (*n* = 23 (13 males and 10 females) from 2 litters), term CS (*n* = 20 (11 males and 9 females) from 2 litters), and preterm CS (*n* = 24 (17 males and 7 females) from 4 litters).

### Cesarean section procedure

Cesarean sections (CS) were performed either at E18 after 6 pm (term CS: maximum 6 h before the estimated time of birth) or at E17 after 7 pm (preterm CS: 6 to 24 h before the estimated time of birth) [25]. CS were performed under sterile conditions using a modification of the procedure described by El-Khodor and Boksa [33] where pregnant dams were euthanized by cervical dislocation to prevent the use of anesthetics. Uterine horns were rapidly isolated from their blood supply (in 20–30 s) and pups were delivered, placed on a heating pad (34–35 °C) for 15 min, and softly massaged by using q-tips to remove the liquid from their lungs and stimulate breathing. Immediately after, CS-delivered pups were given to a surrogate mother that gave birth on the same day.

### Preterm birth procedure

Preterm birth was induced by administering mifepristone treatment (adapted from [34]) at E17 between 1 and 2 am to induce delivery on the following evening. Pregnant mice received a single injection of a 25 μl-dose containing 150 μg of mifepristone (Sigma-Aldrich, MO, USA) dissolved in a vehicle solution containing dimethyl sulfoxide (Sigma-Aldrich, MO, USA) and heated cremophor (Sigma-Aldrich, MO, USA) diluted in sterile NaCl 0.9%. Mice were closely observed the following evening for the onset of labor. Mice born between 5 and 8 pm were used as preterm vaginally-delivered pups.

The effect of the vehicle solution was assessed by injecting pregnant mice at E18 between 7 and 8 am (similar exposure time before birth than for the preterm vaginal group) with a single 25 μl-dose of the solution. No behavioral differences were observed in pups born vaginally or after an injection of the vehicle solution (data not shown).

### Ultrasonic vocalizations

Isolation-induced ultrasonic vocalizations (USVs) were recorded in P9 pups to evaluate early communicative behaviors [25]. Testing was conducted in the morning during the light cycle in a testing room maintained at 21–22 °C. Following a 30-min habituation, pups were isolated one by one from their mother and placed into an isolation box (23 × 28 × 18 cm) located inside a sound-attenuated chamber (54 × 57 × 41 cm; Couldbourn Instruments, PA, USA). USVs were recorded for 3 min using an ultrasound microphone sensitive to frequencies of 10–250 kHz (Avisoft UltraSoundGate condenser microphone capsule CM16/CMPA; Avisoft bioacoustics, Germany) and the Avisoft recorder software (version 4.2) with a sampling rate of 250 kHz in a 16-bit format. Data were transferred to SASLab Pro software (version 5.2; Avisoft bioacoustics, Germany) and a fast Fourier transform (FFT) was conducted to create a spectrogram with a 512 FFT-length, 100% Hamming window, 75% temporal resolution overlap, frequency resolution of 488 Hz, and a sampling frequency of 22,050 Hz. Spectrograms were analyzed for the number of calls, total duration of calls, mean duration of calls and peak frequency.

### Eye opening

The onset of eye opening is a physical milestone that is used as a general guide to assess development. The day of eye opening was controlled twice-daily (at 8 am and 6 pm) starting at P11. The onset of eye opening was marked as the day when the separation of the upper and lower eyelid was present for both eyes.

### Statistics

Analysis was performed using the OriginPro (OriginLab Corporation, MA, USA) and GraphPad Prism 8 (GraphPad Software Inc., CA, USA) software. Data were tested for normality and homoscedasticity before performing statistical analysis. Datasets for the global population were analyzed by one-way ANOVA (when the normality and homoscedasticity assumptions were met), Kruskal-Wallis (when the normality assumption was not met), or Brown-Forsythe ANOVA (when the homoscedasticity assumption was not met). Datasets for the male and female subpopulations were analyzed for multiple comparisons by either the two-tails t-test (when the normality and homoscedasticity assumptions were met), Mann-Whitney test (when the normality assumption was not met) or Welch’s t-test (when the homoscedasticity assumption was not met). For each subpopulation, 6 comparisons were performed: term vaginal vs preterm vaginal, term vaginal vs term CS, term vaginal vs preterm CS, preterm vaginal vs term CS, preterm vaginal vs preterm CS, and term CS vs preterm CS. The Bonferroni correction for multiple comparisons was applied as α = 0.05/number of tests performed. Thus, α was set at 0.0083 (=0.05/6 comparisons) for comparisons within each subpopulation. Between subpopulations, an additional 4 comparisons were evaluated: male term vaginal vs female term vaginal, male preterm vaginal vs female preterm vaginal, male term CS vs female term CS, and male preterm CS vs female preterm CS. The Bonferroni correction was applied with α set at 0.0125 for the comparisons between males and females (=0.05/4 comparisons). Detailed statistics are reported in the Supplementary Materials.

## Results

To better understand the impact of changes in the time and/or mode of birth on early development, we evaluated two distinct patterns through the neonatal period: isolation-induced ultrasonic vocalizations (USVs) and the onset of eye opening. Next, we evaluated whether these parameters could be differently affected in the male and female subpopulations.

**Fig. 1:**
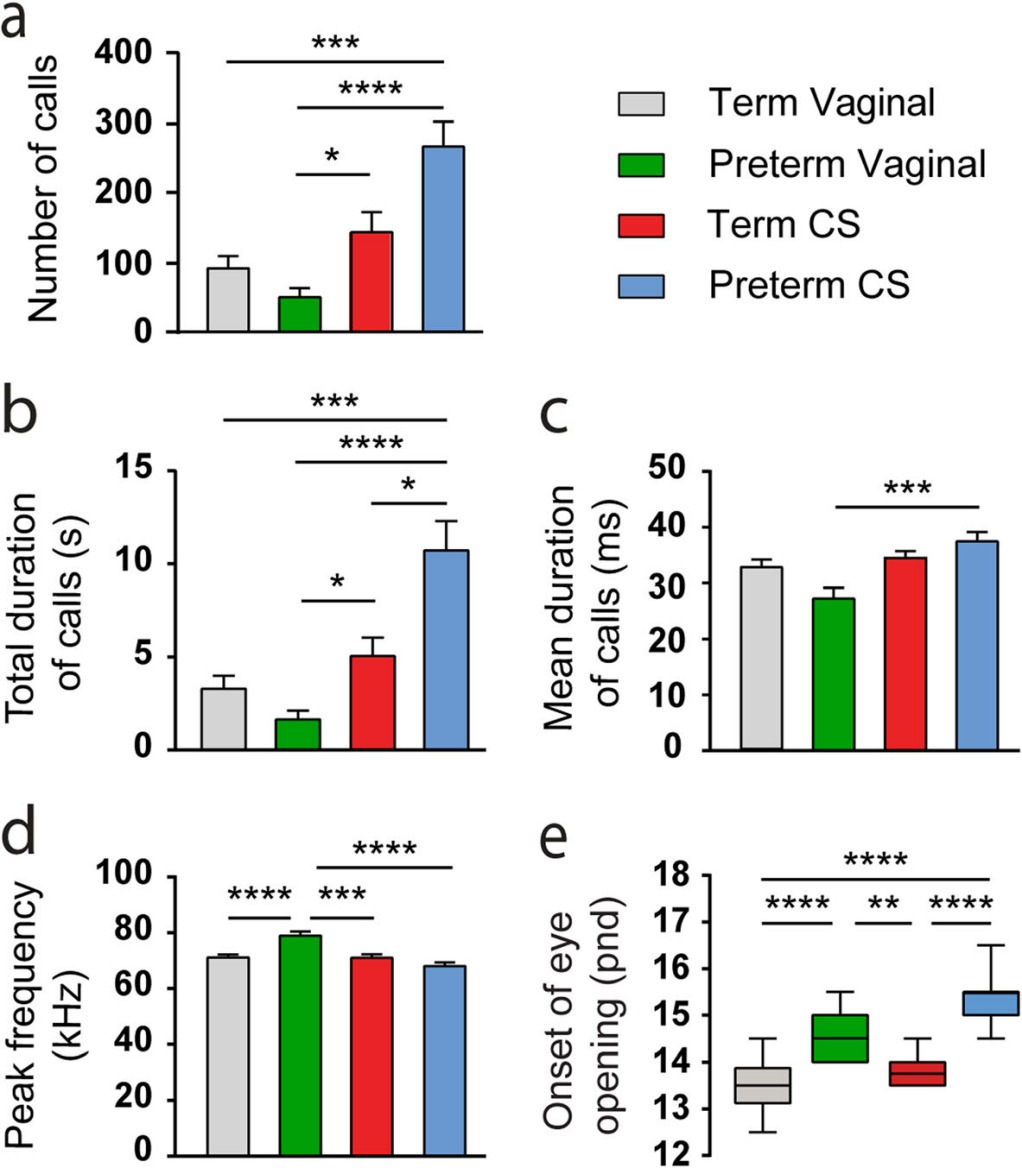
Effect of changes in the time and/or mode of delivery on developmental patterns in the mouse global population. **a**-**d** Influence of the mode and/or time of delivery on USVs in P9 mice for the number of calls (**a**), total duration of calls (**b**), mean duration of calls (**c**), and peak frequency (**d**). **e** Day of eye opening in postnatal days (pnd). Data are presented as mean ± SEM, *p < 0.05, **p < 0.01, ***p < 0.001, ****p < 0.0001. *n* = 36 for vaginal, *n* = 23 for preterm vaginal, *n* = 20 for term CS, and *n* = 24 for preterm CS

In the global population, preterm CS mice emitted significantly more USVs than term vaginal (*p* = 0.0005) and preterm vaginal mice (*p* = 2.01*10^− 5^; Fig. 1a and Supplementary Table 1). The number of calls was also higher for term CS compared with preterm vaginal (*p* = 0.04) but not term vaginal mice (*p* = 0.5534; Fig. 1a and Supplementary Table 1). Likewise, the total duration of the calls emitted was higher for preterm CS mice compared with all groups (*p* = 0.0009 vs term vaginal, *p* = 4.09*10^− 5^ vs preterm vaginal, *p* = 0.0250 vs term CS; Fig. 1b and Supplementary Table 2), and term CS mice called for a longer time than preterm vaginal (*p* = 0.0231) but not term vaginal ones (*p* = 0.6352; Fig. 1b and Supplementary Table 2). Also, the mean duration of calls was lower for preterm vaginal compared with preterm CS (*p* = 0.0005) but not term vaginal mice (*p* = 0.1899; Fig. 1c and Supplementary Table 3), while the peak frequency of these calls was higher for preterm vaginal as compared with term vaginal (*p* = 4.42*10^− 5^), term CS (*p* = 3.47*10^− 4^) and preterm CS mice (*p* = 1.94*10^− 7^; Fig. 1d and Supplementary Table 4). With regards to the onset of eye opening, preterm CS and preterm vaginal mice presented a delay as compared with term vaginal (*p* = 1.10*10^− 14^ and *p* = 3.01*10^− 7^ respectively) and term CS mice (*p* = 4.76*10^− 7^ and *p* = 0.0076 respectively; Fig. 1e and Supplementary Table 5). No difference could be seen in the day of eye opening between preterm CS and preterm vaginal mice (*p* = 0.1699). Thus, our results suggest that in the global population, the number of calls and their total duration are only altered when the time and way of being born are simultaneously changed, peak frequency is only affected by vaginal preterm birth, and the delay in the onset of eye opening is dependent on prematurity solely.

**Fig. 2:**
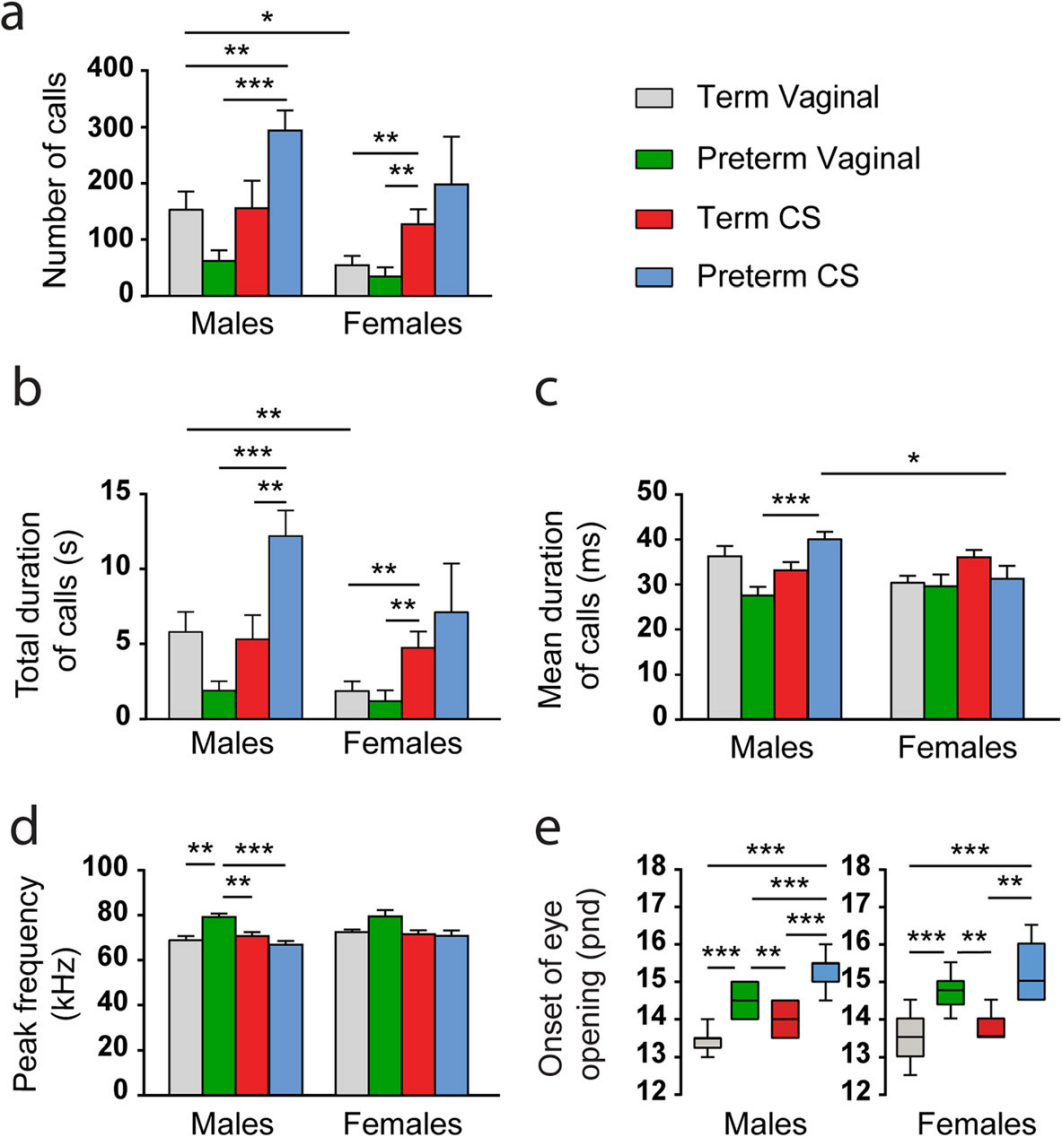
Effect of changes in the time and/or mode of delivery on developmental patterns in the male and female mouse subpopulations. **a**-**d** Influence of the mode and/or time of delivery on USVs in P9 male and female mice for the number of calls (**a**), total duration of calls (**b**), mean duration of calls (**c**), and peak frequency (**d**). **e** Day of eye opening in pnd for the male and female subpopulations. Data are presented as mean ± SEM, *p < 0.0125, **p < 0.0083, ***p < 0.0001. *n* = 13 males, *n* = 23 females for vaginal; *n* = 13 males, *n* = 10 females for preterm vaginal; *n* = 11 males, *n* = 9 females for term CS; and *n* = 17 males, *n* = 7 females for preterm CS

We further assessed whether these parameters were similarly influenced in the male and female subpopulations to uncover possible sex-dependent vulnerabilities. Changes in the mean duration of calls and peak frequency were only found in males, with the mean duration being shorter in preterm vaginal as compared with preterm CS mice (*p* = 3.97*10^− 5^; Fig. 2c and Supplementary Table 8). The peak frequency was higher in preterm vaginal male mice as compared with all groups (*p* = 3.09*10^− 4^ vs term vaginal, *p* = 0.0015 vs term CS, *p* = 5.98*10^− 5^ vs preterm CS; Fig. 2d and Supplementary Table 9). However, the other parameters were affected in distinct ways in both males and females. In males, the mean number of calls was smaller in term and preterm vaginal mice as compared with preterm CS (*p* = 0.0083 and *p* = 6.39*10^− 6^), while in females it was smaller in term and preterm vaginal than in term CS mice (*p* = 0.0036 and *p* = 0.0015; Fig. 2a and Supplementary Table 6). Similarly, in males, the total duration of these calls was longer in mice born preterm by CS than in preterm vaginal (*p* = 1.56*10^− 5^) and term CS mice (*p* = 0.0080). In females, a longer total duration was observed in term CS mice as compared with term vaginal (*p* = 0.0036) and preterm vaginal mice (*p* = 9.74*10^− 4^; Fig. 2b and Supplementary Table 7). Interestingly, in term vaginal mice, the mean number and total duration of calls were gender-dependent (*p* = 0.0111 and *p* = 0.0080 respectively) with males showing higher values than females for both parameters. In contrast, gender differences for the mean duration of calls were only present in the preterm CS group, with males vocalizing for a longer time than females (*p* = 0.0115; Fig. 2a-c and Supplementary Tables 6-8). Regarding the onset of eye opening, similar trends were observed in both the male and female subpopulations (Fig. 2e and Supplementary Table 10), with no difference between them within each group (Supplementary Table 10). However, in males, preterm CS mice opened their eyes later than preterm vaginal mice (*p* = 5.95*10^− 5^), a difference that could not be observed in the female subpopulation (*p* = 0.1205). Thus, our results suggest that these behavioral and developmental patterns, in addition to being distinctly modulated by the mode and/or time of delivery, also underlie a gender-dependent vulnerability in disfavor of males.

## Discussion

Our results show that changes in the time and/or way of being born differentially affect the eye opening developmental milestone and early communications in mice. Specific neonatal USVs alterations were observed in the global population, with the number of calls and total duration of calls being higher in preterm CS mice and the peak frequency being altered only in mice born preterm by vaginal delivery. Distinct behavioral pattern alterations were observed in males and females, with the number of calls and total duration being affected in males of the preterm CS and females of the term CS groups, and the peak frequency being altered only in preterm vaginal males. In addition, a delayed onset of eye opening was observed following changes in the time and/or way of being born, delay that in the male subpopulation was aggravated when both birth changes were present simultaneously.

In rodents, eye opening is a developmental milestone that results from both mechanical and physiological factors consisting in the maturation of the retina, corneal epithelia, and keratinized epidermis and conjunctiva [35, 36], as well as the reorganization of the visual cortex [37–41]. Because children born preterm present visual deficits such as reduced visual acuity and refractive error [27], and our results show that mice born preterm have a delayed onset of eye opening, it would be interesting to evaluate whether the pattern of activity of the visual cortex is altered to elucidate how post-retinal processing might be affected following preterm birth. Future studies should also focus on understanding how CS delivery might further alter these brain patterns of activity.

Ultrasonic vocalizations are communicative behaviors emitted by rodents in various occasions including during mating, aggressive play, and in the presence of predators [42–44]. Neonatal vocalizations which are commonly assessed by separating pups from their mother to elicit stress are proposed to characterize communication deficits, a pattern that is present in numerous neurodevelopmental disorders including Down Syndrome [45] and ASD [46, 47]. In most disease-related studies, tested mice emit fewer calls as compared to controls [42] although this behavioral pattern cannot be generalized. Indeed, female PSD95^+/−^ mice exhibit increased vocalization upon exposure to another mouse [48], while BTBR T + tf/J and SAPAP3-deficient (SAP90/PSD95-associated protein 3) mice pups present a higher number of calls when separated from their mother [49, 50]. Winkler and colleagues suggested that transgenic mice displaying fewer calls may carry a monogenic mutation with restricted and narrow functions, while mutations targeting proteins responsible for the organization of multiple proteins complexes may lead to more intricate behavioral outcomes [48]. Moreover, the expression of sex-specific proteins such as Foxp1 and Foxp2, two proteins involved in the generation and modulation of USVs in mice, may explain our observation of the gender-dependent vulnerability to birth changes [51, 52]. Finally, since the generation and modulation of vocalizations is regulated by the cingulo-periaqueductal and the motor cortex-reticular formation pathways [53, 54], the alterations we observed following preterm vaginal or CS delivery might reflect specific modifications of one of these two networks that would be interesting to address in future studies.

Ultrasonic vocalizations and eye opening involve specific brain regions and functions. Noteworthy, neurodevelopmental disorders present neuronal defects in distinct structures including the amygdala for ASD [55] and prefrontal areas for schizophrenia [56]. We may hypothesize that these defects depend on the modulation of brain development that itself depends on the complex exposure to perinatal insults such as alterations in the way and/or time of being born, hence explaining the disparities reported in epidemiological studies. Thus, our past and present observations further shed light on the intricacy of deviating from vaginal birth at term, substantiating the recent systematic review and meta-analysis of Zhang and colleagues that evaluated the association between cesarean section delivery with the risk of developing psychiatric disorders [57]. In addition, because the fetus has to be prepared for the imminent onset of parturition, some mechanisms must alert the fetal brain to protect itself from any potential harm induced by hypoxic or hypoglycemic insults during labor. Our team previously showed that the abolition of the transient oxytocin-mediated GABA hyperpolarizing shift at birth induces long-term consequences including autistic-like behaviors [58]. Moreover, the analgesic effect mediated by oxytocin and GABA signaling is dampened when babies are born by CS delivery [4, 59]. We can then expect that these adjustments are deficient depending on the way delivery is modified and explain the long-term deleterious consequences reported in some epidemiological studies.

Gender-specific vulnerability should also be carefully considered when studying the influence of birth modifications on early development. Indeed, epidemiological studies on perinatal adverse outcomes [60–62] or the emergence of psychiatric disorders such as ASD [63], schizophrenia [64, 65], and attention deficit hyperactivity disorder [66] have revealed an imbalanced male-to-female ratio with stronger symptoms in males. However, recent work also revealed that females have their own plethora of deleterious manifestations [67–69], warning against the extrapolation of male disease mechanisms to females. Interestingly, our results are in accordance with this view, with males exhibiting stronger symptoms than females, and males and females having dissimilar deficits following birth modifications in behavioral patterns such as the ultrasonic vocalizations.

For these reasons, our results strengthen the idea that reporting in a more systematic manner all perinatal factors during birth is crucial. Indeed, numerous details are often not disclosed including drug dosage information (for analgesics, antalgics, labor induction medications), type of CS performed (elective vs emergency) and gestational length. Moreover, gender-dependent evaluations are necessary as findings reported in the global population might undermine/overestimate the ones found for each specific subpopulation. Thus, exhaustively reporting all factors involved at and around birth could help define better protocols and reduce non-medically indicated interventions by understanding the impact that each deviation from vaginal delivery at term, sole or combined, can have on the fetus’ short and long-term development.

### Limitations

In addition to the issue of the mouse gestational age and its correspondence to humans that has already been addressed in our previous work [25], this short report presents two limitations. The first one concerns the use of surrogate mothers to rear CS-delivered pups and its possible influence on their development. We do not believe this biased our results as term CS mice have previously been shown to present control-like early and long-term features [25]. The second one is focused on the effect of intrauterine exposure to mifepristone on pup’s development. However, Dr. Kawasaki and his team have shown that the in utero injection of mifepristone does not affect pups’ survival rate, suckling behavior, righting reflex, nor body weight [5, 6], therefore suggesting that the effects observed in the mifepristone-treated group reflect changes due to a premature birth.

## Supplementary information

**Additional file 1: Supplementary Table 1.** Mean number of calls recorded at P9. **Supplementary Table 2.** Total duration of calls recorded at P9. **Supplementary Table 3.** Mean duration of calls recorded at P9. **Supplementary Table 4.** Peak frequency of calls recorded at P9. **Supplementary Table 5.** Onset of eye opening. **Supplementary Table 6.** Mean number of calls recorded at P9 in males and females. **Supplementary Table 7.** Total duration of calls recorded at P9 in males and females. **Supplementary Table 8.** Mean duration of calls recorded at P9 in males and females. **Supplementary Table 9.** Peak frequency of calls recorded at P9 in males and females. **Supplementary Table 10.** Onset of eye opening in males and females.

## Data Availability

Mice used in this study are commercially available. The datasets used for this study are available from the corresponding author upon reasonable request.

## Abbreviations

ASD: Autism spectrum disorders
CS: Cesarean section
FFT: Fast Fourier transform
GABA: Gamma-aminobutyric acid
USVs: Ultrasonic vocalizations
