# Supplementary material for: Alteration in the time and/or mode of delivery differentially modulates early development in mice
Source: Mol Brain. 2020 Mar 9;13:34. doi: 10.1186/s13041-020-00578-5 (PMC7063737; doi:10.1186/s13041-020-00578-5)
Supplement: Supplementary file 1 — Additional file 1: Supplementary Table 1. Mean number of calls recorded at P9. Supplementary Table 2. Total duration of calls recorded at P9. Supplementary Table 3. Mean duration of calls recorded at P9. Supplementary Table 4. Peak frequency of calls recorded at P9. Supplementary Table 5. Onset of eye opening. Supplementary Table 6. Mean number of calls recorded at P9 in males and females. Supplementary Table 7. Total duration of calls recorded at P9 in males and females. Supplementary Table 8. Mean duration of calls recorded at P9 in males and females. Supplementary Table 9. Peak frequency of calls recorded at P9 in males and females. Supplementary Table 10. Onset of eye opening in males and females. [file 13041_2020_578_MOESM1_ESM.docx]

***Supplementary Material***

**Alteration in the time and/or mode of delivery differentially modulates early development in mice**

Morgane Chiesa^1^, Diana C. Ferrari^1*^, Yehezkel Ben-Ari^1*^

^1^Neurochlore, Ben-Ari Institute of Neuroarcheology (IBEN), Fundamental Research Department, Marseille, France.

***Correspondence:**

Yehezkel Ben-Ari and Diana C. Ferrari

ben-ari@neurochlore.fr; ferrari@neurochlore.fr

**Supplementary Tables**

**Supplementary Table 1.** Mean number of calls recorded at P9. Statistics are presented by the Brown-Forsythe ANOVA with the Tamhane’s T2 multiple comparisons post-hoc test.

| Figure **1a** | Number of calls, mean ± SEM | |
| --- | --- | --- |
| Term vaginal | 89.78 ± 17.58 | |
| Preterm vaginal | 49.74 ± 13.05 | |
| Term CS | 143.00 ± 28.96 | |
| Preterm CS | 266.13 ± 35.46 | |
| Comparisons | Brown-Forsythe ANOVA | Tamhane’s T2 test |
| Term vaginal vs preterm vaginal | F*(3, 63.28)=13.93  **p=4.47*10^-7^** | p=0.3644 |
| Term vaginal vs term CS |  | p=0.5534 |
| Term vaginal vs preterm CS |  | **p=0.0005** |
| Preterm vaginal vs term CS |  | **p=0.0400** |
| Preterm vaginal vs preterm CS |  | **p=2.01*10^-5^** |
| Term CS vs preterm CS |  | p=0.0600 |

**Supplementary Table 2.** Total duration of calls recorded at P9. Statistics are presented by the Brown-Forsythe ANOVA with the Tamhane’s T2 multiple comparisons post-hoc test.

| Figure **1b** | Total duration of calls (s), mean ± SEM | |
| --- | --- | --- |
| Term vaginal | 3.29 ± 0.70 | |
| Preterm vaginal | 1.60 ± 0.47 | |
| Term CS | 5.05 ± 0.99 | |
| Preterm CS | 10.72 ± 1.57 | |
| Comparisons | Brown-Forsythe  ANOVA | Tamhane’s T2 test |
| Term vaginal vs preterm vaginal | F*(3, 54.79)=15.04  **p=2.92*10^-7^** | p=0.2668 |
| Term vaginal vs term CS |  | p=0.6352 |
| Term vaginal vs preterm CS |  | **p=0.0009** |
| Preterm vaginal vs term CS |  | **p=0.0231** |
| Preterm vaginal vs preterm CS |  | **p=4.09*10^-5^** |
| Term CS vs preterm CS |  | **p=0.0250** |

**Supplementary Table 3.** Mean duration of calls recorded at P9. Statistics are presented by the one-way ANOVA with the Tukey’s multiple comparisons post-hoc test.

| Figure **1c** | Mean duration of calls (ms), mean ± SEM | |
| --- | --- | --- |
| Term vaginal | 32.57 ± 1.36 | |
| Preterm vaginal | 28.49 ± 1.55 | |
| Term CS | 34.48 ± 1.24 | |
| Preterm CS | 37.49 ± 1.64 | |
| Comparisons | One-way  ANOVA | Tukey post-hoc test |
| Term vaginal vs preterm vaginal | F(3, 97)=5.86  **p=0.0010** | p=0.1899 |
| Term vaginal vs term CS |  | p=0.7983 |
| Term vaginal vs preterm CS |  | p=0.0677 |
| Preterm vaginal vs term CS |  | p=0.0516 |
| Preterm vaginal vs preterm CS |  | **p=0.0005** |
| Term CS vs preterm CS |  | p=0.5428 |

**Supplementary Table 4.** Peak frequency of calls recorded at P9. Statistics are presented by the one-way ANOVA with the Tukey’s multiple comparisons post-hoc test.

| Figure **1d** | Peak frequency (kHz), mean ± SEM | |
| --- | --- | --- |
| Term vaginal | 71.12 ± 1.05 | |
| Preterm vaginal | 79.32 ± 1.47 | |
| Term CS | 71.06 ± 1.24 | |
| Preterm CS | 68.02 ± 1.37 | |
| Comparisons | One-way  ANOVA | Tukey post-hoc test |
| Term vaginal vs preterm vaginal | F(3, 97)=13.15  **p=2.82*10^-7^** | **p=4.42*10^-5^** |
| Term vaginal vs term CS |  | p=0.9999 |
| Term vaginal vs preterm CS |  | p=0.2634 |
| Preterm vaginal vs term CS |  | **p=3.47*10^-4^** |
| Preterm vaginal vs preterm CS |  | **p=1.94*10^-7^** |
| Term CS vs preterm CS |  | p=0.3963 |

**Supplementary Table 5.** Onset of eye opening. Statistics are presented by the Kruskal-Wallis with the Dunn’s multiple comparisons post-hoc test.

| Figure **1e** | Onset of eye opening, mean ± SEM | |
| --- | --- | --- |
| Term vaginal | 13.50 ± 0.07 | |
| Preterm vaginal | 14.61 ± 0.10 | |
| Term CS | 13.84 ± 0.09 | |
| Preterm CS | 15.31 ± 0.11 | |
| Comparisons | Kruskal-Wallis | Dunn post-hoc test |
| Term vaginal vs preterm vaginal | H(3)=74.22  **p=5.34*10^-16^** | **p=3.01*10^-7^** |
| Term vaginal vs term CS |  | p=0.5543 |
| Term vaginal vs preterm CS |  | **p=1.10*10^-14^** |
| Preterm vaginal vs term CS |  | **p=0.0076** |
| Preterm vaginal vs preterm CS |  | p=0.1699 |
| Term CS vs preterm CS |  | **p=4.76*10^-7^** |

**Supplementary Table 6.** Mean number of calls recorded at P9 in males and females. The Bonferroni correction for multiple comparisons was applied with α=0.0083 to compare within each subpopulation, and α=0.0125 to compare between males and females.

| Figure **2a** | Number of calls, mean ± SEM | |
| --- | --- | --- |
| Male term vaginal | 153.08 ± 32.40 | |
| Male preterm vaginal | 61.85 ± 19.09 | |
| Male term CS | 155.91 ± 48.92 | |
| Male preterm CS | 294.12 ± 35.58 | |
| Female term vaginal | 54.00 ± 16.88 | |
| Female preterm vaginal | 34.00 ± 16.65 | |
| Female term CS | 127.22 ± 26.72 | |
| Female preterm CS | 198.14 ± 85.10 | |
| Comparisons | Statistics | |
| Male term vaginal vs male preterm vaginal | Mann-Whitney | U=123.00, Z=1.95  p=0.0489 |
| Male term vaginal vs male term CS |  | U=77, Z=0.29  p=0.7637 |
| Male term vaginal vs male preterm CS | Two-tails t-test | t(28)= -2.84  **p=0.0083** |
| Male preterm vaginal vs male term CS | Welch t-test | t(13.03)= -1.79  p=0.0965 |
| Male preterm vaginal vs male preterm CS |  | t(23.90)= -5.75  **p=6.39*10^-6^** |
| Male term CS vs male preterm CS | Mann-Whitney | U=47.00, Z= -2.16  p=0.0287 |
| Female term vaginal vs female preterm vaginal |  | U=134.00, Z=0.73  p=0.4686 |
| Female term vaginal vs female term CS |  | U=36.00, Z= -2.81  **p=0.0036** |
| Female term vaginal vs female preterm CS | Welch t-test | t(6.48)= -1.66  p=0.1440 |
| Female preterm vaginal vs female term CS | Mann-Whitney | U=8.00, Z= -2.98  **p=0.0015** |
| Female preterm vaginal vs female preterm CS | Welch t-test | t(6.46)= -1.89  p=0.1037 |
| Female term CS vs female preterm CS |  | t(7.19)= -0.80  p=0.4520 |
| Male term vaginal vs female term vaginal | Mann-Whitney | U=225.50, Z=2.49  **p=0.0111** |
| Male preterm vaginal vs female preterm vaginal |  | U=85.50, Z=1.24  p=0.2139 |
| Male term CS vs female term CS |  | U=41.00, Z= -0.61  p=0.5516 |
| Male preterm CS vs female preterm CS |  | U=75.50, Z=0.98  p=0.3226 |

**Supplementary Table 7.** Total duration of calls recorded at P9 in males and females. The Bonferroni correction for multiple comparisons was applied with α=0.0083 to compare within each subpopulation, and α=0.0125 to compare between males and females.

| Figure **2b** | Total duration of calls (s), mean ± SEM | |
| --- | --- | --- |
| Male term vaginal | 5.79 ± 1.34 | |
| Male preterm vaginal | 1.91 ± 0.63 | |
| Male term CS | 5.31 ± 1.61 | |
| Male preterm CS | 12.20 ± 1.71 | |
| Female term vaginal | 1.88 ± 0.65 | |
| Female preterm vaginal | 1.20 ± 0.72 | |
| Female term CS | 4.74 ± 1.08 | |
| Female preterm CS | 7.12 ± 3.24 | |
| Comparisons | Statistics | |
| Male term vaginal vs male preterm vaginal | Welch t-test | t(16.96)=2.62  p=0.0179 |
| Male term vaginal vs male term CS | Mann-Whitney | U=79.00, Z=0.41  p=0.6905 |
| Male term vaginal vs male preterm CS | Two-tails t-test | t(28)= -2.80  p=0.0091 |
| Male preterm vaginal vs male term CS | Welch t-test | t(12.99)= -1.97  p=0.0711 |
| Male preterm vaginal vs male preterm CS |  | t(20.08)= -5.65  **p=1.56*10^-5^** |
| Male term CS vs male preterm CS | Mann-Whitney | U=38.00, Z= -2.59  **p=0.0080** |
| Female term vaginal vs female preterm vaginal |  | U=133.00, Z=0.69  p=0.4994 |
| Female term vaginal vs female term CS |  | U=36.00, Z= -2.81  **p=0.0036** |
| Female term vaginal vs female preterm CS | Welch t-test | t(6.49)= -1.59  p=0.1596 |
| Female preterm vaginal vs female term CS | Mann-Whitney | U=7.00, Z= -3.06  **p=9.74*10^-4^** |
| Female preterm vaginal vs female preterm CS | Welch t-test | t(6.60)= -1.78  p=0.1201 |
| Female term CS vs female preterm CS |  | t(7.35)= -0.70  p=0.5074 |
| Male term vaginal vs female term vaginal | Mann-Whitney | U=229.00, Z=2.60  **p=0.0080** |
| Male preterm vaginal vs female preterm vaginal |  | U=81.00, Z=0.96  p=0.3434 |
| Male term CS vs female term CS |  | U=42.00, Z= -0.53  p=0.6027 |
| Male preterm CS vs female preterm CS |  | U=84.00, Z=1.52  p=0.1297 |

**Supplementary Table 8.** Mean duration of calls recorded at P9 in males and females. The Bonferroni correction for multiple comparisons was applied with α=0.0083 to compare within each subpopulation, and α=0.0125 to compare between males and females.

| Figure **2c** | Mean duration of calls (ms), mean ± SEM | | |
| --- | --- | --- | --- |
| Male term vaginal | 36.26 ± 2.32 | | |
| Male preterm vaginal | 27.58 ± 1.88 | | |
| Male term CS | 33.16 ± 1.82 | | |
| Male preterm CS | 40.04 ± 1.67 | | |
| Female term vaginal | 30.39 ± 1.53 | | |
| Female preterm vaginal | 29.59 ± 2.62 | | |
| Female term CS | 36.08 **±** 1.59 | | |
| Female preterm CS | 31.28 ± 2.86 | | |
| Comparisons | | Statistics | |
| Male term vaginal vs male preterm vaginal | | Two-tails t-test | t(23)=2.88  p=0.0085 |
| Male term vaginal vs male term CS | |  | t(22)=1.02  p=0.3171 |
| Male term vaginal vs male preterm CS | |  | t(28)= -1.36  p=0.1855 |
| Male preterm vaginal vs male term CS | |  | t(21)= -2.13  p=0.0453 |
| Male preterm vaginal vs male preterm CS | |  | t(27)= -4.90  **p=3.97*10^-5^** |
| Male term CS vs male preterm CS | |  | t(26)= -2.70  p=0.0119 |
| Female term vaginal vs female preterm vaginal | |  | t(30)=0.28  p=0.7807 |
| Female term vaginal vs female term CS | |  | t(29)= -2.18  p=0.0374 |
| Female term vaginal vs female preterm CS | |  | t(27)= -0.28  p=0.7802 |
| Female preterm vaginal vs female term CS | |  | t(17)= -2.06  p=0.0553 |
| Female preterm vaginal vs female preterm CS | |  | t(15)= -0.43  p=0.6742 |
| Female term CS vs female preterm CS | |  | t(14)=1.56  p=0.1419 |
| Male term vaginal vs female term vaginal | |  | t(33)=2.20  p=0.0347 |
| Male preterm vaginal vs female preterm vaginal | |  | t(20)= -0.64  p=0.5324 |
| Male term CS vs female term CS | |  | t(18)= -1.18  p=0.2536 |
| Male preterm CS vs female preterm CS | |  | t(22)=2.76  **p=0.0115** |

**Supplementary Table 9.** Peak frequency of calls recorded at P9 in males and females. The Bonferroni correction for multiple comparisons was applied with α=0.0083 to compare within each subpopulation, and α=0.0125 to compare between males and females.

| Figure **2d** | Peak frequency (kHz), mean ± SEM | |
| --- | --- | --- |
| Male term vaginal | 68.87 ± 1.87 | |
| Male preterm vaginal | 79.19 **±** 1.52 | |
| Male term CS | 70.71 ± 1.77 | |
| Male preterm CS | 66.90 ± 1.62 | |
| Female term vaginal | 72.45 ± 1.20 | |
| Female preterm vaginal | 79.49 ± 2.78 | |
| Female term CS | 71.50 ± 1.80 | |
| Female preterm CS | 70.76 ± 2.46 | |
| Comparisons | Statistics | |
| Male term vaginal vs male preterm vaginal | Two-tails  t-test | t(23)= -4.24  **p=3.09*10^-4^** |
| Male term vaginal vs male term CS |  | t(22)= -0.71  p=0.4879 |
| Male term vaginal vs male preterm CS | Mann-Whitney | U=132.00, Z=0.88  p=0.3851 |
| Male preterm vaginal vs male term CS | Two-tails t-test | t(21)=3.66  **p=0.0015** |
| Male preterm vaginal vs male preterm CS | Mann-Whitney | U=186.00, Z=3.70  **p=5.98*10^-5^** |
| Male term CS vs male preterm CS |  | U=131.00, Z=1.74  p=0.0817 |
| Female term vaginal vs female preterm vaginal | Two-tails t-test | t(30)= -2.74  p=0.0102 |
| Female term vaginal vs female term CS |  | t(29)=0.43  p=0.6687 |
| Female term vaginal vs female preterm CS |  | t(27)=0.67  p=0.5078 |
| Female preterm vaginal vs female term CS |  | t(17)=2.35  p=0.0312 |
| Female preterm vaginal vs female preterm CS |  | t(15)=2.23  p=0.0418 |
| Female term CS vs female preterm CS |  | t(14)=0.25  p=0.8059 |
| Male term vaginal vs female term vaginal |  | t(33)= -1.69  p=0.1000 |
| Male preterm vaginal vs female preterm vaginal |  | t(20)= -0.10  p=0.9229 |
| Male term CS vs female term CS |  | t(18)= -0.31  p= 0.7594 |
| Male preterm CS vs female preterm CS | Mann-Whitney | U=39.00, Z= -1.27  p=0.2094 |

**Supplementary Table 10.** Onset of eye opening in males and females. The Bonferroni correction for multiple comparisons was applied with α=0.0083 to compare within each subpopulation, and α=0.0125 to compare between males and females.

| Figure **2e** | Onset of eye opening, mean ± SEM | |
| --- | --- | --- |
| Male term vaginal | 13.46 ± 0.09 | |
| Male preterm vaginal | 14.54 ± 0.13 | |
| Male term CS | 13.91 ± 0.13 | |
| Male preterm CS | 15.35 ± 0.10 | |
| Female term vaginal | 13.52 ± 0.10 | |
| Female preterm vaginal | 14.70 ± 0.15 | |
| Female term CS | 13.78 ± 0.12 | |
| Female preterm CS | 15.21 ± 0.31 | |
| Comparisons | Statistics | |
| Male term vaginal vs male preterm vaginal | Mann-Whitney | U=5.00, Z= -4.18  **p=4.04*10^-6^** |
| Male term vaginal vs male term CS |  | U=33, Z= -2.42  p=0.0188 |
| Male term vaginal vs male preterm CS |  | U=0.00, Z= -4.70  **p=1.67*10^-8^** |
| Male preterm vaginal vs male term CS |  | U=117.50, Z=2.73  **p=0.0071** |
| Male preterm vaginal vs male preterm CS |  | U=25.00, Z= -3.71  **p=5.95*10^-5^** |
| Male term CS vs male preterm CS |  | U=1.50, Z= -4.38  **p=3.73*10^-7^** |
| Female term vaginal vs female preterm vaginal |  | U=9.50, Z= -4.22  **p=2.59*10^-6^** |
| Female term vaginal vs female term CS |  | U=72.50, Z= -1.37  p=0.1929 |
| Female term vaginal vs female preterm CS |  | U=1.50, Z= -3.95  **p=3.93*10^-6^** |
| Female preterm vaginal vs female term CS |  | U=83.50, Z=3.19  **p=9.74*10^-4^** |
| Female preterm vaginal vs female preterm CS | Two-tails t-test | t(15)= -1.65  p=0.1205 |
| Female term CS vs female preterm CS | Welch t-test | t(7.88)= -4.37  **p=0.0025** |
| Male term vaginal vs female term vaginal | Mann-Whitney | U=136.50, Z= -0.45  p=0.7518 |
| Male preterm vaginal vs female preterm vaginal |  | U=54.00, Z= -0.69  p=0.5606 |
| Male term CS vs female term CS |  | U=57.50, Z=0.62  p=0.6449 |
| Male preterm CS vs female preterm CS | Welch t-test | t(7.40)=0.43  p=0.6797 |
